# A Case of Suspected Carpal Tunnel Syndrome: Ultrasonography Prior to the Scalpel

**DOI:** 10.7759/cureus.65823

**Published:** 2024-07-31

**Authors:** Ujjawal Roy, Achal Kumar Srivastava, Michael S Cartwright, Ajay Panwar, Pratik Shahil

**Affiliations:** 1 Department of Neurology, Roy Neurocare Centre, Ranchi, IND; 2 Department of Neurology, Pulse Super Speciality Hospital, Ranchi, IND; 3 Neurology, All India Institute of Medical Sciences, New Delhi, New Delhi, IND; 4 Department of Neurology, Wake Forest University School of Medicine, Winston-Salem, USA; 5 Neurology, Rotary Ambala Cancer and General Hospital, Amballa Cantt., IND

**Keywords:** rheumatoid arthritis, neuromuscular ultrasound, point of care ultrasonography, ultrasonography, carpal tunnel syndrome

## Abstract

Carpal tunnel syndrome (CTS) is the most prevalent focal mononeuropathy worldwide and is traditionally diagnosed based on clinical history, examination, and nerve conduction studies. Surgical release is the gold standard in cases where conservative management fails; however, it is prudent to obtain imaging before planning such intervention. We present the case of a 47-year-old woman who presented with typical symptoms of CTS, which was confirmed with nerve conduction studies and was being considered for carpal tunnel release surgery. Her history and laboratory studies revealed rheumatoid arthritis and subsequent ultrasonography showed swelling of the median nerve just proximal to the transverse carpal ligament at the level of pronator quadratus muscle. The possibility of a nerve sheath tumour or tenosynovitis with edematous fascicles of the median nerve was considered, and a decision was taken to give a short course of prednisolone 1 mg/kg, to which she clinically responded and the repeat ultrasonography showed near complete resolution of the focal nerve enlargement. This case emphasizes the role of point-of-care neuromuscular ultrasound (NMUS) in identifying the underlying cause of CTS and validates NMUS as a powerful tool in reaching a comprehensive diagnosis in entrapment neuropathies and it should be incorporated into the routine protocol of diagnosis of these disorders.

## Introduction

Carpal tunnel syndrome (CTS) is the most common entrapment neuropathy encountered in clinical practice. [1] The hallmark of the syndrome is pain, numbness, and tingling in the median nerve distribution. If not properly and timely managed, the condition may progress to permanent sensory loss, clumsiness, weakness, and thenar atrophy [2].

Typically, CTS is diagnosed on the basis of electrodiagnostic testing, in the form of nerve conduction studies (NCS), which show only sensory abnormalities in cases of mild CTS, whereas in moderate and severe CTS, both sensory and motor abnormalities are noted [3]. In mild cases, initially, conservative management is tried which includes splinting, corticosteroids, physical therapy, therapeutic ultrasound, and drugs such as pregabalin/gabapentin. Other options that have been tried by clinicians include steroid injections and ultrasound-guided hydrodissection. Surgical management is recommended in cases of failed conservative measures for mild and moderate cases and for the most severe cases [4]. However, failure of carpal tunnel release surgery is not extremely rare, and therefore, understanding the exact diagnosis and pathophysiology is of prime importance when planning for surgical management [5].

Unfortunately, NCS does not directly reveal underlying pathology. Hence, bedside tools such as nerve ultrasonography have been increasingly used to assess the pathophysiology and mechanism of injury of the nerve, which can immensely help the neurologist and surgeon [6]. Here we present a unique ultrasonographic finding in a suspected case of CTS which proved to be instrumental in the precise diagnosis and successful management of the patient.

## Case presentation

A 47-year-old woman with hypothyroidism was referred by orthopaedic surgery for evaluation. She had a one-year history of paresthesias, numbness, and pain in the thumb, index, and middle fingers. Four months prior, NCS showed prolonged distal sensory and motor latencies of the median nerve (motor latency = 4.9 ms, normal < 4 ms; sensory latency = 4.1 ms, normal < 3.5 ms). She was previously prescribed gabapentin, physical therapy, and splinting with mild relief in symptoms, but for two months prior to our appointment, the symptoms had worsened. Repeat NCS showed no change. Laboratory parameters were normal except for a high cyclic citrullinated peptide (CCP) (509.7, normal <5.0), C-reactive protein (CRP) (7.0, normal <0.3), and erythrocyte sedimentation rate (ESR) (60, normal <20).

Ultrasonography of the median nerve was performed using a Venue Go™ R4 (GE HealthCare Technologies, Inc., Chicago, Illinois, United States) with a 4-20 MHz linear transducer. Ultrasound showed a distinct hypoechoic lesion in the lateral aspect of the median nerve, with a cross-sectional area of 3 mm^2^ at the distal wrist crease (Video 1). On power Doppler, vascularity was increased in the periphery of the lesion (Video 2). 

**Video 1:** Ultrasonography shows a distinct hypoechoic lesion in the lateral aspect of the median nerve encircled in blue

**Video 2:** Ultrasonography shows increased vascularity in the periphery of the lesion on power Doppler

We determined the possible causes of the lesion to be a peripheral nerve tumour or focal inflammation within the nerve. In view of elevated CRP, ESR, and anti-CCP levels, we tried oral prednisolone at 1 mg/kg dose for 30 days and repeated the imaging. Upon follow-up, the patient's symptoms had resolved. Repeat ultrasonography showed near complete resolution of the lesion (Video 3). She was prescribed a slow taper of prednisolone, initiated on methotrexate, and a close follow-up was arranged. It was ultimately thought that her condition was most likely secondary to an inflammatory lesion within the median nerve, related to rheumatoid arthritis.

**Video 3:** Repeat ultrasonography after one month shows near complete resolution of the lesion

## Discussion

As with all of medicine, understanding the exact underlying pathophysiology is of prime importance, especially for preparing management protocols and optimizing outcomes. Approximately 5% of people suffer from CTS worldwide, especially those between 40 and 60 years [1]. There are several risk factors for this condition including menopause, obesity, diabetes, kidney failure, hypothyroidism, use of oral contraceptives, pregnancy, and congestive heart failure. Other conditions predisposing to CTS include rheumatoid arthritis and posttraumatic arthritis [7]. Our patient had several predisposing factors for CTS including age, female gender, and hypothyroidism.

In this case, the point-of-care ultrasound revealed an atypical finding, with swelling on the radial side of the median nerve, which could be from an inflammatory nerve lesion, described in the literature and termed as rheumatoid neuropathy by some authors [8,9]. Although we had kept the possibility of nerve sheath tumours such as Schwannoma or neurofibroma in the differential, these were less likely given the resolution of the lesion following oral corticosteroid treatment. Less likely considerations included a malignant nerve sheath tumour, lipofibromatous hamartoma, intraneural perineurioma, intraneural lipoma, neurothekeoma, and plexiform neurofibroma. Non-neural lesions such as a lipoma, ganglion cyst, and fibroma were also unlikely [10], as the lesion appeared to have a neural origin.

As evident in our case, ultrasonography was the key to reaching an accurate diagnosis and potentially avoiding surgery. Several previous studies have highlighted that diagnosis of CTS based on only NCS has false positive and false negative results, and ultrasonography has a definitive role in the comprehensive approach to this disorder [11]. However, ultrasonography is not always part of the routine diagnosis of CTS [12]. Future studies are warranted to inform management paradigms both in cases of idiopathic CTS as well as in those with secondary causes such as inflammatory arthritis.

## Conclusions

This case emphasizes the role of point-of-care neuromuscular ultrasound in identifying the aetiology and location of the lesion in median mononeuropathies, which can aid in the accurate diagnosis of these disorders. An accurate diagnosis leads to appropriate treatment considerations, including whether surgery is the right approach. Its cost-effectiveness, feasibility to be used at bedside and in outpatient clinics with efficient use of time, and accessibility and portability make it an ideal modality for evaluating individuals with suspected CTS. Hence, point-of-care ultrasound can be a powerful tool in reaching an accurate diagnosis and can aid in the preparation of comprehensive management paradigms in entrapment neuropathies, especially CTS, and should be incorporated into the routine protocol for these disorders.
